# The Investigation of Therapeutic Implications of Mast Cell Stabilizer Cromolyn Sodium on Cholestasis and Cholestatic Pruritus in Experimental Cholestasis

**DOI:** 10.5152/tjg.2022.21744

**Published:** 2023-01-01

**Authors:** Hanife Yurdakul Ertan, Günnur Özbakış Dengiz, Figen Barut, Yonca Yılmaz Ürün, Murat Can, Shemsu Umer Hussen, Yücel Üstündağ

**Affiliations:** 1 Department of Internal Medicine, Zonguldak Bülent Ecevit University Faculty of Medicine, Zonguldak, Turkey; 2 Department of Medical Pharmacology, Zonguldak Bülent Ecevit University Faculty of Medicine, Zonguldak, Turkey; 3 Department of Pathology, Zonguldak Bülent Ecevit University Faculty of Medicine, Zonguldak, Turkey; 4 Department of Gastroenterology and Hepatology, Eskişehir City Hospital, Turkey; 5 Department of Medical Biochemistry, Zonguldak Bülent Ecevit University Faculty of Medicine, Zonguldak, Turkey; 6 Department of Medical Pharmacology, Zonguldak Bülent Ecevit University Faculty of Medicine, Zonguldak, Turkey; 7 Department of Gastroenterology and Hepatology, Zonguldak Bülent Ecevit University Faculty of Medicine, Zonguldak, Turkey

**Keywords:** Autotaxin, cholestasis, cromolyn sodium, mast cells, pruritus

## Abstract

**Background:**

Relevant studies have indicated that hepatic mast cells may have potential roles in the progression of cholestasis and cholestasis-induced itch. We aimed to compare the effects of cromolyn sodium and other medications on cholestatic pruritus, serum biochemistry, histamine, total bile acids, autotaxin, liver histopathology, and mast cell distribution in tissues in an experimental cholestasis model conducted by bile duct ligation.

**Methods:**

Rats received the determined treatment consecutively for 10 days in addition to bile duct ligation. On the 5th and 10th days of the experiment, the rats’ itching behaviors were observed for 5 minutes. After 10 days, blood and tissue samples were taken.

**Results:**

Significant decreases in serum histamine and autotaxin levels, plasma total bile acids, total bilirubin, and biliary enzymes were reported only in cromolyn sodium-treated rats compared to the control group. In immunohistochemistry of the liver samples, the peribiliary mast cells stained positive for autotaxin. Except for bile duct infarctus, all histopathological findings of cholestasis significantly improved only in cromolyn sodium-treated and sertraline-treated rats. The liver and peritoneal mast cells significantly decreased only in cromolyn sodium-treated rats compared to the control group. On the 10th day of the experiment, the mean duration of itching was significantly lower in all groups, except for naloxone- and ondansetron-treated rats.

**Conclusion:**

Cromolyn sodium has promising antipruritic efficacy and provides biochemical and histopathological recovery of the relevant parameters of cholestasis induced by bile duct ligation. For the first time in the literature, we showed that peribiliary mast cells can produce autotaxin, which is a very important pruritogenic signal in the setting of cholestasis.

**Main Points**

- Hepatic mast cells may have potential roles in cholestasis-induced itch.
- Autotaxin (ATX) correlates with the pruritus and can be used to monitor the treatment response.
- Cromolyn sodium has promising antipruritic efficacy.
- Cromolyn sodium provided biochemical recovery in the parameters of cholestasis.
- Cromolyn sodium improved hepatobiliary injury via decreasing ATX and histamine.

## Introduction

Aside from their notorious role in allergic and inflammatory reactions, mast cells (MCs) are implicated in liver pathologies, such as hepatitis, fibrosis, and cirrhosis and allograft rejection after liver transplantation.^1,2^ Recent reports also indicated its role in cholangiopathy-associated liver injury and pruritus. For example, MC quantity as well as plasma histamine levels have been shown to increase in patients with primary biliary cirrhosis (PBC) and primary sclerosing cholangitis (PSC). An increase in MCs positive for basic fibroblast growth factor (bFGF) and other profibrogenic mediators, such as heparin, histamine, and tryptase, which were found to accumulate around the proliferating bile ducts and in the portal triad, has been linked to the progression of fibrosis and ductular injury in various cholangiopathies, including PBC.^3^ Furthermore, there is clear evidence of elevated histamine concentration in bile duct ligation (BDL) models of cholestasis, activation of MCs by bile acids, increased skin MC accumulation in jaundiced animals, and decreased biliary hyperplasia in cholestatic rodents after MC stabilizer (cromolyn sodium [CROM] administration).^1^ It is clear that histamine has a potential role in cholestatic itch, and the MC stabilizer CROM might have benefits with regard to the control of pruritus, regression of ductular injury, and liver fibrosis.^1,4^

In recent years, the neuronal activator lysophosphatidic acid (LPA) and its forming unit autotaxin (ATX) have been shown to be key factors in the cholestasis-associated pruritogenic signal cascade, and serum ATX levels have been reported to correlate with the level of pruritus.^5^ Thus, ATX levels can potentially be used to monitor the response to conventional medical treatment of cholestatic itching. Ursodeoxycholic acid (UDCA), cholestyramine, antihistamines, rifampin, and opioid receptor antagonists have been used to treat patients with cholestatic pruritus with some success.^6-8^ Nevertheless, all these drugs sometimes fail to achieve control of itching that can be generalized, disabling, and refractory to their combination. To the best of our knowledge, MC stabilizers have not been investigated in the field of obstructive cholestasis-associated itching.

In this study, we aimed to investigate and compare the effects of CROM and various other therapeutic agents on serum biochemistry, serum histamine, total bile acids, ATX levels, liver histopathology, and MC distribution in the liver, and skin and peritoneum in experimental cholestasis induced by BDL in rats. We also aimed to scrutinize the efficacy of CROM on cholestatic itching and compare its effectiveness with other therapeutic agents used in this area.

## Materials and Methods

This trial was approved by the Animal Experiments Local Ethics Committee of the Zonguldak Bülent Ecevit University Medical Faculty, and the animals were housed in an experimental animal research laboratory.

### Animals and Cholestasis Induction

**Figure 1.**
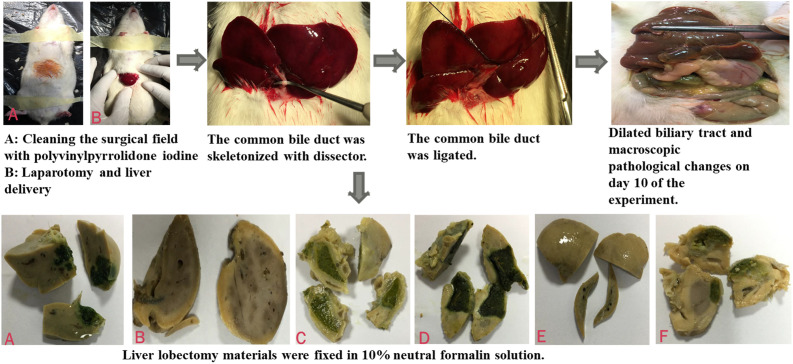
The study chart of the bile duct ligation and lobectomy is seen. (A) Lobectomy sections of control group. (B) Lobectomy sections of sham group. (C) Lobectomy sections of BDL+NAL. (D) Lobectomy sections of BDL+OND. (E-F) Lobectomy sections of BDL-CROM.

Sixty-nine Sprague–Dawley male rats weighing 193-296 g were randomly divided into 8 groups. The rats were housed at appropriate room temperature and light. They were fed free-access standard rat feed and water. Oral consumption was stopped 12 hours before the experiment. We weighed the rats preoperatively, and 25 mg/kg intraperitoneal (i.p.) thiopenthal was applied for general anesthesia. Next, the rats were fixed to the operation table under appropriate lighting. The operation region was shaved and cleaned with a 10% polyvinylpyrrolidone iodine antiseptic solution, and a 2-3 cm midline incision was applied to enter the abdominal cavity. The supraduodenal common bile duct (CBD) was sutured with 5/0 silk sutures in all groups, except the sham group, after removal of the liver in an appropriate way, and finally, total extrahepatic obstruction was achieved. After the operation, the rat’s abdominal cavity was closed by suturing with 3/0 silk suture. Daily wound care was performed, and the wound region was checked for wound infection regularly. The determined treatment was applied everyday consecutively for 10 days after the surgery. The CBD was mobilized but not ligated during the laparotomy, and no medical treatment was given to the sham group (n = 9). The control group (n = 12) received i.p. saline as the treatment. BDL+ chlorpheniramine maleate (CPM) group (n = 8) received i.p. CPM, BDL+ sertraline (SERT) group (n = 8) oral SERT, BDL+ ondansetron (OND) group (n = 8) i.p. OND, BDL+UDCA group (n = 7) oral UDCA, BDL+ naloxone (NAL) group (n = 8) i.p. NAL, and BDL+CROM group (n = 9) i.p. CROM. On the 5th and 10th days of the experiment, subjects were observed for 5 minutes in terms of itching behavior. After 10 days, the rats were weighed and blood samples were taken under general anesthesia. Finally, tissue (skin-subcutaneous, peritoneal membrane, and liver) samples were taken for histopathological studies after sacrification. All BDL and lobectomy steps are shown in Figure 1.

### Evaluation of Blood Samples

Biochemical analysis (alanine aminotransferase [ALT], aspartate aminotransferase [AST], alkaline phosphatase [ALP], gamma-glutamyl transpeptidase [GGT], total and direct bilirubin) was performed by serum spectrophotometric methods. Total bile acids were quantified by a colorimetric assay, which was based on an enzymatic cycling method in the presence of nicotinamide adenine dinucleotide and chromophores, and the detection limit of the test was 1 μM. Histamine and ATX levels were measured by enzyme-linked immunosorbent assay (ELISA). Autotaxin levels were quantified by ELISA. The ATX coefficient of variability intertest and intratest was <12%, and the ATX sensitivity was 0.064 ng/mL. The histamine coefficient of variability was <12%, and the histamine sensitivity was 0.54 ng/mL.

### Evaluation of Histopathologic Findings

All tissue samples were fixed in 10% neutral formalin solution and embedded in paraffin blocks. Serial slices of 4-5 μm thickness were obtained from paraffin blocks. All sections were deparaffinized and stained with hematoxylin and eosin for morphological examination. Liver injury due to BDL was evaluated semiquantitatively for the typical histopathological findings by light microscopy by a blinded pathologist. Histopathologic findings were as follows: portal inflammation, lobular inflammation, bile duct proliferation, and necrosis, which were scored as follows: 0: no, 1: mild, 2: intermediate, 3: manifest, and 4: severe. Additionally, bile-induced infarction was scored according to the degree of damage: 0: no infarction, 1: mild infarction, 2: intermediate infarction, 3: manifest infarction, and 4: severe infarction. Fibrosis was graded as follows: 0: no fibrosis, 1: portal enlargement, 2: septal formation, 3: manifest bridging fibrosis, and 4: cirrhosis. Microscopic images were obtained from each study group. Tissue samples were stained with immunohistochemical stains to show MCs. Slices of 4-5 μm thickness were taken from the paraffin blocks. Sections were deparaffinized and stained with “MC tryptase” mouse monoclonal primary antibody in an automatic immunohistochemical staining device after preapplication with citrate pH:7.0 by the microwave heating method. Reactive cells were counted in every high-power area (HPA) to determine MCs by a blinded pathologist.

## Immunohistochemical Methods for Autotaxin Expression in Peribiliary Mast Cells

Formalin-fixed paraffin-embedded liver sections were stained with immunohistochemistry. Four-micrometer-thick sections were taken from the liver tissue, and the sections were deparaffinized. Liver sections were analyzed with anti-ENNPP2/ATX antibody using an automated immunohistochemical staining device, and a human tonsil was used as a positive control. The sections were pretreated using heat-mediated antigen retrieval with sodium citrate buffer (pH = 6, epitope retrieval solution 1) for 20 minutes, and they were incubated with anti-ENNPP2/ATX antibody, 1 μg/mL, for 15 minutes at room temperature and detected using an horseradish peroxidase (HRP)-conjugated compact polymer system. 3,3’-Diaminobenzidine (DAB) was used as the chromogen. The section was then counterstained with hematoxylin and mounted with entellan. Reactive MCs in the portal area were counted in every HPA by a blinded pathologist.

### Statistical Analysis

Statistical analysis was performed using Statistical Package for the Social Sciences version 20.0 software (IBM Corp.; Armonk, NY, USA). Numerical variables are expressed as the mean ± standard error of the mean. Differences between groups were analyzed by Kruskal–Wallis variance analysis, followed by the Mann–Whitney *U* test for pairwise comparison of groups. Differences were considered statistically significant at *P* < .05.

## Results

Clinical findings of cholestasis, such as jaundice, dark urine, and icteric plasma, were observed in the rats undergoing BDL. Weight loss was detected in the control, BDL+CPM, and BDL+NAL groups.

**Figure 2.**
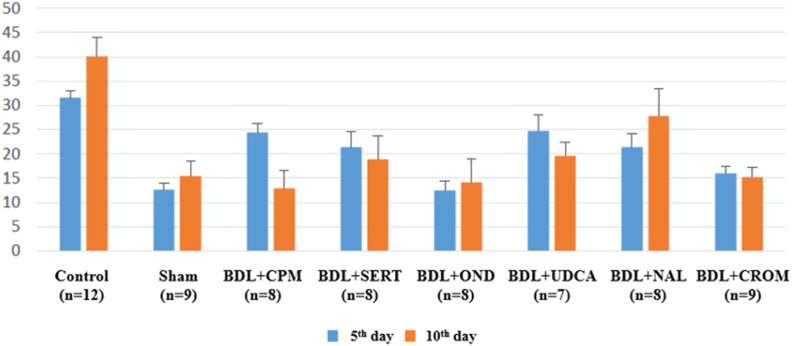
The main duration of itching time for the whole groups on the fifth and tenth days is demonstrated.

On the fifth day of the experiment, the mean duration of itching in the control group was 31.58 ± 1.42 s/5-min observation period, which was significantly higher than that in the sham group (12.67 ± 1.29 s/5 min) (*P* < .001). In all study groups, the main duration of itching ranged from 21.38 ± 3.11 s/5 min to 24.71 ± 3.23 s/5 min, which was significantly lower than that of the control group (*P* < .05). On the 10th day of the experiment, the main duration of itching, which ranged from 14.13 ± 4.79 s/5 min to 27.71 ± 5.6 s/5 min, was significantly lower in all groups than in the control group (*P* < .05). However, it was higher in the control group, sham group, BDL+OND group, and BDL+NAL group on the 10th day of the experiment than on the 5th day, and it was lower in the BDL+CPM, BDL+SERT, BDL+UDCA, and BDL+CROM groups on the 10th day of the study than on the 5th day (Figure 2).

### Biochemical Parameters

Plasma AST, ALT, ALP, GGT, total and direct bilirubin levels, serum histamine, and ATX levels significantly increased in the control group compared with the sham group as a result of cholestasis (*P* < .05). Plasma total bile acid levels increased in the control group compared to the sham group, but it was not statistically significant. Nevertheless, plasma total bile acid levels decreased in all study groups compared with the control group, and this reduction was statistically significant only in the BDL+CROM group (*P =* .001).

Serum histamine levels were lowest in the BDL+CROM and BDL+CPM groups (*P =* .001 and *P =* .01, respectively) compared to the control group. Serum ATX levels also decreased significantly in the BDL+CROM group and BDL+NAL group compared to the control group (*P =* .003 and *P =* .012, respectively), and the lowest value was found in the BDL+CROM group.

Plasma AST levels were lowest in the BDL+SERT and BDL+NAL groups compared to the control group (*P =* .037 and *P =* .022, respectively). Plasma ALT levels decreased in all groups compared to the control group, and the reductions in ALT and AST levels in the BDL+CROM group were not found to be statistically significant.

**Figure 3.**
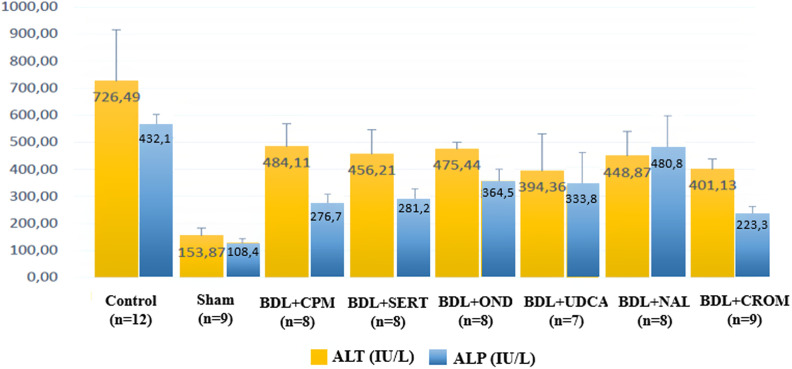
The ALT and ALP levels of all groups are depicted. ALT, alanine aminotransferase; ALP, alkaline phosphatase.

**Figure 4.**
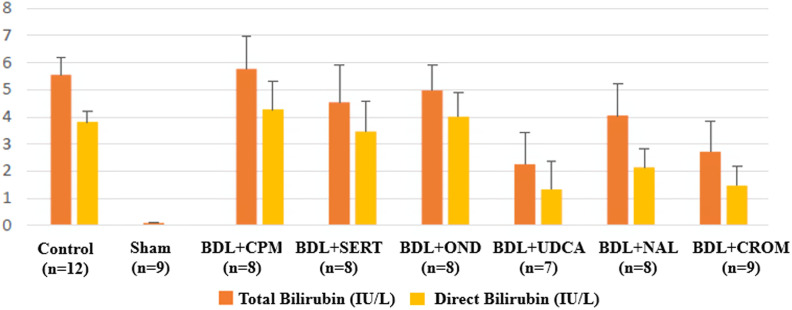
The total and direct bilirubin levels of all the groups are shown.

Plasma ALP, GGT, and total and direct bilirubin levels decreased significantly in the BDL+CROM group (*P* < .05). Plasma GGT and total and direct bilirubin significantly decreased in the BDL+UDCA group (*P* < .05). The lowest values of ALP and GGT were in the BDL+CROM, and the lowest values of total and direct bilirubin were in the BDL+UDCA group among the rest of the treatment groups. Additionally, plasma ALP levels decreased significantly in both the BDL+CPM and the BDL+SERT groups (*P =* .017 and *P =* .023, respectively), but the plasma GGT and total and direct bilirubin levels did not significantly decrease in these groups (Figures 3 and 4). There were no statistically significant differences among the biochemical parameters in the BDL+OND group compared to the control group, whereas the most prominent reduction was detected in the BDL+CROM group. The alterations in biochemical parameters are shown in Table 1.

### Histopathologic Findings

Liver damage due to BDL was evaluated on a light microscope. Significant differences were determined for portal inflammation, lobular inflammation, bile duct proliferation, necrosis, and fibrosis between the study groups, whereas bile duct infarct was not found to be significantly different between the groups (*P =* .104) (Table 2).

Obvious liver damage due to BDL was demonstrated in the control group, and only mild lobular and portal inflammation was detected in the sham group. There was a statistically significant difference between the control group and the sham group in terms of portal inflammation, lobular inflammation, bile duct proliferation, necrosis, and fibrosis (*P* < .05). All these findings were significantly apparent in the control group. Portal enlargement, septal formation, and manifest bridging necrosis and fibrosis were found in the control group but not in the sham group. None of the study groups developed cirrhosis. The fibrosis scores significantly decreased in study groups (*P* < .05). Although the reduction in the portal and lobular inflammation was considerably observed in all study groups, the decrease in portal inflammation was not statistically significant in the BDL+NAL and BDL+UDCA groups (*P* > .05). In addition, neither the reduction in portal inflammation nor lobular inflammation was statistically significant in the BDL+UDCA group (*P* > .05).

**Figure 5.**
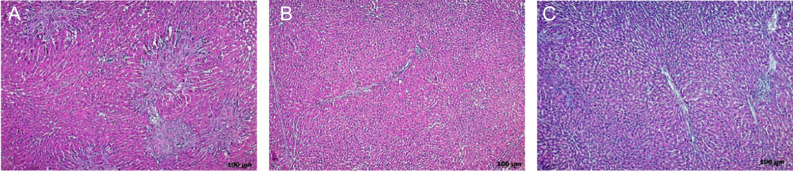
A comparison of liver histopathology in the BDL+CROM group with sham and control groups. (Light microscopic images of the liver tissue with hematoxylin and eosin staining are seen). (A) Liver damage caused by bile duct ligation in the control group. (B) Mild lobular and portal inflammation in liver tissue in the sham group. (C) Light microscopic images of the liver tissue in the BDL+CROM group with hematoxylin-eosin staining. BDL, bile duct ligation; CROM, cromolyn sodium.

A statistically significant reduction in bile duct proliferation was only seen in the BDL+CROM group (*P* < .001) (Figure 5). A statistically significant improvement in terms of necrosis was only seen in the BDL+CROM and BDL+SERT groups (*P =* .042 and *P =* .015, respectively). There were no specific histopathological findings in the skin and peritoneal tissues in the study groups.

**Figure 6.**
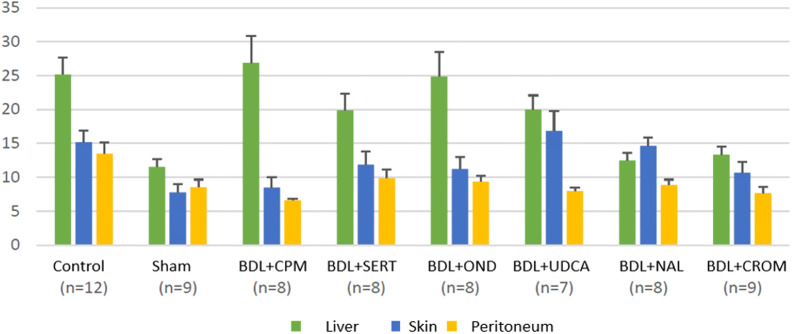
Mast cell counts in the liver, the skin, and the peritoneum.

**Figure 7.**
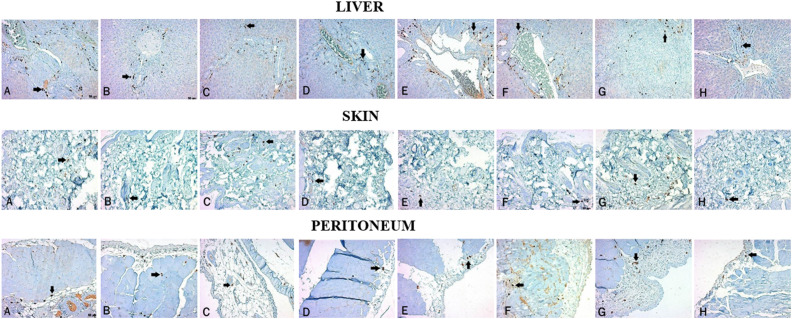
Mast cell distribution of the liver, skin, and peritoneum with immunohistochemical staining is demonstrated (mast cells are shown with arrows). (A) Control group, (B) sham group, (C) BDL+CPM group, (D) BDL+SERT group, (E) BDL+OND group, (F) BDL+UDCA group, (G) BDL+NAL group, and (H) BDL+CROM group. BDL, bile duct ligation; CROM, cromolyn sodium; CPM, chlorpheniramine maleate; SERT, sertraline; OND, ondansetron; UDCA, ursodeoxycholic acid; NAL, naloxone.

**Figure 8.**
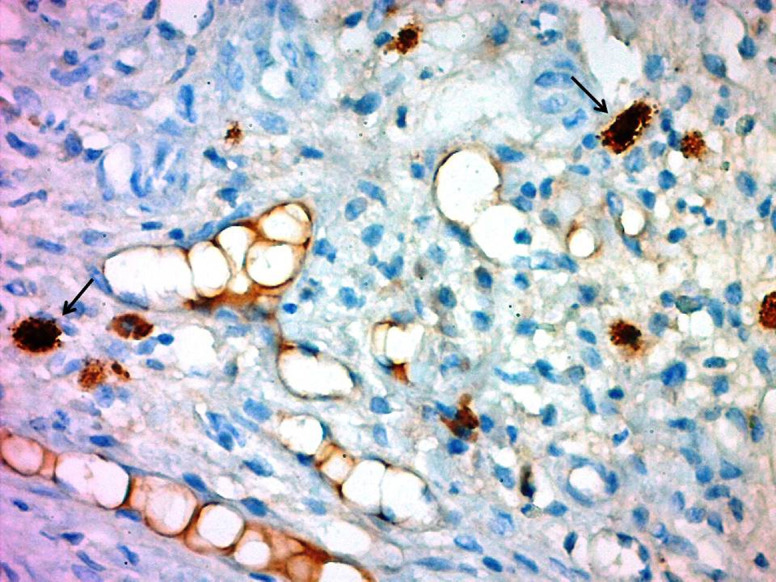
Granulated peribiliary mast cells displaying ATX expression (black arrow: mast cell). ATX, autotaxin.

The presence of MCs was detected in liver, skin, and peritoneal specimens with immunohistochemical staining. There were statistically significant differences in terms of liver, skin, and peritoneal MC counts between the study groups *(P* < .001, *P =* .008, and *P =* .002, respectively). Mast cells in both the liver and the peritoneum were significantly decreased in the BDL+CROM group compared to the control group (*P =* .003, *P =* .004, respectively), whereas the decrease in skin MCs was not statistically significant (*P* = .09). The liver MC count was significantly lower in the BDL+CROM (*P =* .003) and BDL+NAL groups (*P =* .003) than in the control group. The skin MC count was significantly lower in the BDL+CPM group (*P =* .022). Although the skin MC count decreased in the BDL+CROM group, this reduction was not statistically significant (*P =* .09). The peritoneal MC count was significantly lower in the BDL+CROM (*P =* .004), BDL+CPM (*P* < .001), BDL+UDCA (*P =* .009), and BDL+NAL (*P =* .016) groups than in the control group. Additionally, there was no statistically significant reduction in liver, skin, or peritoneal MC count in the BDL+SERT and BDL+OND groups. In the BDL+UDCA group, only the peritoneal MC count significantly decreased (*P =* .009). Mean MC counts of the tissues are shown in Figure 6. The MC distribution of the liver, skin, and peritoneum with immunohistochemical staining is shown in Figure 7. We also clearly show that peribiliary MCs expressed ATX on immunohistochemical analysis (Figure 8).

## Discussion

Mast cells were once considered important immune cells only for allergy-mediated events and wound healing processes. However, it has been revealed that these cells contribute to many autoimmune, inflammatory, infectious, and other diseases in many organ systems, including the liver.^9,10^ Mast cells have also been implicated in various liver pathologies, including hepatic fibrosis, hepatitis, liver regeneration, allograft rejection, and liver carcinogenesis.^3^ In particular, we have vast data about the role of MCs in cholangiopathies, such as PBC and PSC. Hepatic MC expression and quantity have been shown to increase in PBS and PSC. They also have important remodeling actions on bile ducts during the progression of PBC and PSC via overexpression of bFGF and histamine.^11,12^ Histamine has been shown to stimulate biliary growth via autocrine mechanisms, and the MC stabilizer CROM has the ability to induce cholangiocyte apoptosis.^1^ Thus, many studies so far provide evidence that MCs may be a therapeutic target for the treatment of cholangiopathies.

We also know the potential of MCs to influence the pathology of obstructive cholangitis and the development of chronic pruritus in this setting. Patients with cholestasis and pruritus had peripheral neuroinflammation, which is recognized by an increase in dermal MC quantity. MCs activate protein-activated receptor 2, which leads to TRPV1 sensitization.^13^ Other than conventional agents used in the control of cholestatic pruritus, such as antihistamines, UDCA, OND, opioids, and serotonin agonists, the pharmacological modulation of upregulated MCs by CROM may have profuse effects on both the pathophysiology of cholestatic pruritus and the lessening of hepatobiliary injury induced by bile duct obstruction. Thus, we aimed to compare CROM with 5 other conventional agents used to control pruritus together with the hepatobiliary effects of these agents.

**Figure 9.**
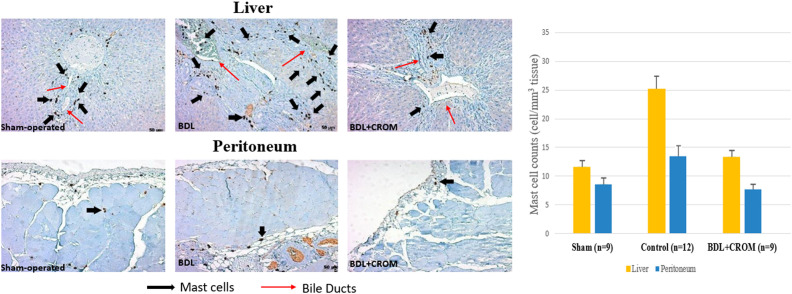
The peribiliary distribution of mast cells and changes of their counts in the BDL+CROM group compared to sham and control groups are seen. BDL, bile duct ligation; CROM, cromolyn sodium.

In our study, we have clear evidence of the increase in MC quantity in the liver, skin, and peritoneum 10 days after BDL in the control group. This increase was also proven by other animal studies that showed a startling increase in liver MCs on day 4 after BDL and gradual escalation in their numbers peaking on the 14th day.^14^ Likewise, skin MC counts at the dermis–epidermis junction were reported to increase 21 days after BDL in another report.^15^ We also noticed significantly increased serum histamine, ATX, and total bile acid levels after BDL in the control group. Although histamine is accepted as a weak mediator of cholestatic pruritus, serum ATX activity correlates well with the degree of pruritus and forms the bulk of LPA, a potent itch neuronal activator.^16^ When we introduced an MC stabilizer in the present study, we found that the liver and peritoneal MC quantity significantly decreased (Figure 9). Moreover, within all the study groups, only the BDL+ CROM group developed a significant decrease in both serum histamine and ATX concentrations. We also know that a subpopulation of CD203c-positive MCs in the gastrointestinal tract was reported to show positive staining for intracellular ATX/lysoPLD by flow cytometry. Moreover, a significant level of ATX/lysoPLD release was detected in the culture supernatants of human MCs by Western blot analysis.^17^ It has already been proven that liver MCs can release various mediators, not only histamine but also heparin, interleukins, cytokines, growth factors, including bFGF, and numerous vasoactive substances.^18^ Thus, there may be a potential link between liver/biliary MCs and ATX production in the setting of cholestasis. We believe the source of elevated circulating ATX activity in the rats with cholestasis in our study can be the peribiliary MCs, as the peribiliary MCs showed positive staining for intracellular ATX on immunohistochemistry of liver samples.

On histopathology, we noticed that MCs gather mostly around the proliferating bile ducts, not in the periphery of the liver or around hepatocytes, which was consistent with the existing literature.^19-21^ We believe that this finding is very important, as MCs directly impact the biliary response following injury. In rat models of BDL, peribiliary MCs were shown to become activated by accumulating bile acids after BDL.^22^ Furthermore, endotoxemia, which occurs consistently after biliary obstruction, was defined to be one of the factors responsible for MC activation in this setting of jaundice.^23^ Furthermore, MC-deficient mice develop much less biliary hyperplasia and liver fibrosis after BDL.^14^ We can say that MCs have a deleterious role in hepatobiliary damage by releasing numerous mediators into the local environment. This paracrine effect can be modified by using an MC stabilizer, as happened in our study, which showed that the BDL+CROM group developed the least hepatobiliary damage after BDL among the other study groups. The most dramatic decrease in biliary hyperplasia was depicted in this group. This was in parallel with previous literature indicating that MC stabilization with CROM induces inhibition of cholangiocyte proliferation, even leading to cholangiocyte apoptosis.^1,21^ Additionally, after 10 days of CROM administration, liver sections showed significantly decreased fibrosis. This may be explained by interpreting the results of another report indicating evidence of MC production of extracellular matrix rich in collagen and laminin when stimulated by bile acids.^24^ Furthermore, other potential antifibrotic effects of CROM were deciphered by a fascinating report that indicated its inhibitory effects overall on stellate cell collagen production ability, transforming growth factor (TGF)-related mesenchymal transformation, and hepatocyte aging.^25^ Moreover, only the BDL+CROM group had a significant decrease in biliary enzymes, such as ALP and GGT, in the present study. Serum bilirubin levels also significantly lessened in this group, although this was more apparent in UDCA-treated rats. Rats that were given UDCA after BDL had lower ALP and GGT levels, but this decrease was not statistically significant. Thus, our study confirms that MCs can be paracrine regulators of hepatobiliary injury.

We also believe that MC activation is a generalized phenomenon in biliary obstruction, given that both dermal and peritoneal MCs were found to increase in quantity in our control group compared to the sham group. Increased dermal MCs have been reported previously both in animal models of cholestasis and in patients with cholestasis and pruritus.^15,26^ It is obvious that dermal MCs may also have a contributory role in promoting pruritus in cholestatic patients by secreting histamine. In our study, we clearly demonstrated decreased quantities of MCs in 3 different locations in the rat body, liver, peritoneum, and skin under treatment with the MC stabilizer CROM. We believe that CROM is highly effective in controlling MCs everywhere in the body; thus, it decreased serum histamine levels much more than conventional antihistaminic CPM in this study. Cromolyn sodium administration also resulted in decreased ATX levels in our study. One report in the literature indicated that ATX/LPD triggers MC histamine and cytokine production.^27,28^ Therefore, we believe that MC stabilizers theoretically have more potency than conventional antihistamines for the control of pruritus in the setting of cholestasis. However, we observed similar rates of success of all the agents in the study groups with regard to itching behavior duration on the 5th and 10th days of the experiment. We believe that this observation method of itching behavior remained subjective and cannot be used for one-to-one comparisons of the potency of antipruritic activity in the study groups.

This study has some limitations. First, the number of rats in the study is insufficient to reach strong results. Second, it was necessary to observe the itching behaviors of the rats at more frequent intervals and for a longer time to evaluate the anti-pruritic efficacy. Last, the lack of subtyping of mast cells in the skin, liver, and peritoneum samples was one of the limitations of our study.

In conclusion, this study pinpointed these MCs as essential regulators of pathogenic processes of obstructive cholestasis and associated pruritus. Systemic MC activation occurs in the setting of bile duct obstruction, and targeting therapy against activation of MCs via CROM demonstrated multiple beneficial effects, such as regression of hepatobiliary injury and control of pruritus, possibly by decreasing various mediators, including ATX and histamine. It is obvious that CROM should be investigated in the treatment algorithm of patients with obstructive jaundice and chronic pruritus. Nevertheless, the extent to which MC and ATX interactions and the subtype of MCs have a dominant role in specific hepatobiliary diseases and cholestasis-associated pruritus is still unknown. Further studies are warranted to investigate the full magnitude of the role of MCs in this setting.

## Figures and Tables

**Table 1.** The Comparison of Biochemical Parameters of the Control Group and Study Groups

**Groups**	**Total Bile Acids (μM)**	**Histamine (ng/mL)**	**Autotaxin** **(ng/mL) **	**ALP (IU/L)**	**GGT** **(IU/L) **	**AST** **(IU/L) **	**ALT** **(IU/L) **	**Total Bilirubin (mg/dL)**	**Direct Bilirubin (ng/mL)**
**Control (n = 12)**	6.89 ± 0.29	9.35 ± 0.18	15.49 ± 0.13	432.1 ± 33.4	60.17 ± 13.7	97.4 ± 18.09	726.4 ± 189.2	5.56 ± 0.63	3.81 ± 0.4
**Sham (n = 9)**	**5.96 ± 0.35**	7.57 ± 0.31	**14.86 ± 0.09**	**108.4 ± 15.8**	0.11 ± 0.11	**34.42 ± 6.24**	**153.8 ± 27.9**	**0.09 ± 0.03**	**0.02 ± 0.0**
**BDL+CPM (n = 8)**	6.28 ± 0.41	**8.16 ± 0.33**	15.24 ± 0.17	**276.7 ± 50.8**	52.25 ± 23.19	74.59 ± 15.77	484.1 ± 85.4	5.77 ± 1.19	4.27 ± 1.06
**BDL+SERT (n = 8)**	6.19 ± 0.59	9.40 ± 0.29	15.22 ± 0.14	**281.2 ± 39.5**	14 ± 3.9	**51.45 ± 8.56**	456.2 ± 88.7	4.54 ± 1.37	3.47 ± 1.12
**BDL+OND (n = 8)**	6.42 ± 0.12	9.17 ± 0.25	15.23 ± 0.11	364.5 ± 42.5	33 ± 8.73	72.35 ± 5.42	475.4 ± 23.7	4.98 ± 0.92	4.01 ± 0.87
**BDL+UDCA (n = 7)**	6.44 ± 0.63	9.20 ± 0.21	15.44 ± 0.21	333.8 ± 119.3	**11.57 ± 5.79**	66.39 ± 19.12	394.3 ± 136.9	**2.26 ± 1.18**	1.33 ± 1.04
**BDL+NAL (n = 8)**	6.03 ± 0.38	8.68 ± 0.58	**15.02 ± 0.12**	480.8 ± 119.6	**12.57 ± 5.03**	**52.4 ± 11.19**	448.8 ± 90.9	4.05 ± 1.17	2.14 ± 0.67
**BDL+CROM (n = 9)**	**5.28 ± 0.21**	**8.07 ± 0.11**	**14.61 ± 0.42**	**223.3 ± 33.4**	**6.89 ± 2.46**	70.41 ± 5.72	401.1 ± 36.4	**2.72 ± 1.13**	**1.47 ± 0.7**

The biochemical parameters in the control, sham, and study groups 10 days after bile duct ligation in rats are seen.

The study groups were compared with the control group.

Bold values are statistically significant (*P* < .05) (Mann–Whitney *U* test).

ALP, alkaline phosphatase; GGT, gamma-glutamyl transpeptidase; ALT, alanine aminotransferase; AST, aspartate aminotransferase.

**Table 2.** The Liver Histopathological Findings in the Control and Study Groups

**Groups**	**Portal Inflammation**	**Lobular Inflammation**	**Bile Duct Proliferation **	**Necrosis**	**Fibrosis **	**Bile Duct Infarctus **
**Control (n = 12)**	3.67 ± 0.19	3.17 ± 0.21	4.00 ± 0.00	1.83 ± 0.41	2.50 ± 0.15	0.67 ± 0.36
**Sham (n = 9)**	**0.67 ± 0.17**	**0.78 ± 0.15**	**0.00 ± 0.00**	**0.00 ± 0.00**	**0.00 ± 0.00**	0.00 ± 0.00
**BDL+CPM (n = 8)**	**2.63 ± 0.46**	**1.75 ± 0.25**	3.63 ± 0.26	1.00 ± 0.33	**1.75 ± 0.16**	0.13 ± 0.13
**BDL+SERT (n = 8)**	**2.25 ± 0.45**	**1.75 ± 0.41**	**2.63 ± 0.50**	**0.38 ± 0.18**	**1.25 ± 0.25**	0.00 ± 0.00
**BDL+OND (n = 8)**	**2.38 ± 0.18**	**1.75 ± 0.16**	3.63 ± 0.26	1.25 ± 0.31	**2.00 ± 0.00**	0.50 ± 0.50
**BDL+UDCA (n = 7)**	2.86 ± 0.46	2.29 ± 0.36	3.57 ± 0.43	1.29 ± 0.52	**2.00 ± 0.00**	0.00 ± 0.00
**BDL+NAL (n = 8)**	3.00 ± 0.46	**2.00 ± 0.27**	3.25 ± 0.49	1.63 ± 0.50	**1.75 ± 0.16**	1.38 ± 0.68
**BDL+CROM (n = 9)**	**2.00 ± 0.29**	**2.11 ± 0.31**	**2.56 ± 0.41**	**0.67 ± 0.24**	**1.67 ± 0.29**	0.00 ± 0.00

The liver histopathological findings in the control, sham, and study groups 10 days after bile duct ligation in rats are documented.

The study groups are compared with the control group and bold values are statistically significant (*P* < .05).
